# Supplementary material for: Real-time tracking reveals catalytic roles for the two DNA binding sites of Rad51
Source: Nat Commun. 2020 Jun 11;11:2950. doi: 10.1038/s41467-020-16750-3 (PMC7289862; doi:10.1038/s41467-020-16750-3)
Supplement: Supplementary file 1 — Supplementary Information [file 41467_2020_16750_MOESM1_ESM.pdf]

Supplementary information for

Real-time tracking reveals catalytic roles for the two DNA binding sites of Rad51

By Ito et al.

Supplementary Materials including

Supplementary Tables 1-10

Supplementary Figures 1- 6

Supplementary Table 1. ATPase activities (k_{cat} [min^{-1}]) of wild-type and mutant Rad51 proteins

	WT	Rad51-L1	Rad51-L2	Rad51-S2
Rad51 only	0.163 ± 0.027	0.0344 ± 0.0081	0.133 ± 0.017	0.0962 ± 0.014
+ ssDNA	0.178 ± 0.0083	0.0790 ± 0.0081	0.107 ± 0.0068	0.178 ± 0.022
+ S5S1 + ssDNA	0.322 ± 0.019	0.321 ± 0.034	0.231 ± 0.0055	0.308 ± 0.016
+ dsDNA	0.196 ± 0.0051	0.0925 ± 0.0058	0.205 ± 0.043	0.138 ± 0.027
+ S5S1 + dsDNA	0.212 ± 0.011	0.106 ± 0.0087	0.242 ± 0.022	0.149 ± 0.027
+ ssDNA + dsDNA	0.197 ± 0.022	0.118 ± 0.011	0.185 ± 0.0089	0.192 ± 0.017
+ S5S1 + ssDNA + dsDNA	0.309 ± 0.023	0.265 ± 0.019	0.275 ± 0.013	0.279 ± 0.013

Each value reflects the average of three experiments. ± indicates standard deviation. S5S1, Swi5-Sfr1.

Supplementary Table 2. Reaction rate and equilibrium constants obtained from Fig. 2a

	$k_1 \times 10^5$ (M^{-1} s^{-1})	$k_{-1} \times 10^{-2}$ (s^{-1})	$K_1 \times 10^6$ (M^{-1})	$k_2 \times 10^{-3}$ (s^{-1})	$k_{-2} \times 10^{-3}$ (s^{-1})	K_2	$k_3 \times 10^{-3}$ (s^{-1})	$k_{-3} \times 10^5$ (M^{-1} s^{-1})	$K_3 \times 10^{-8}$ (M)
WT	1.86 ± 0.11	6.83 ± 0.53	2.73 ± 0.10	15.1 ± 4.3	35.9 ± 4.4	0.415 ± 0.64	1.54 ± 0.25	0.563 ± 0.28	3.26 ± 1.8
WT+S5S1	1.80 ± 0.081	6.93 ± 0.63	2.60 ± 0.15	18.3 ± 3.0	13.9 ± 2.0	1.31 ± 0.091	3.85 ± 0.10	0.106 ± 0.022	37.3 ± 7.6
Rad51-L2	4.38 ± 0.055	16.6 ± 2.2	2.64 ± 0.031	0.420 ± 0.072	0.218 ± 0.067	2.05 ± 0.68	N.D.	N.D.	N.D.
Rad51-L2+S5S1	3.01 ± 0.39	11.1 ± 1.1	2.71 ± 0.23	4.51 ± 0.70	4.67 ± 1.1	0.990 ± 0.19	3.68 ± 1.0	0.234 ± 0.076	16.7 ± 6.5
Rad51-S2	0.0100 ± 0.0031	0.0630 ± 0.0038	1.58 ± 0.43	N.D.[a]	N.D.[a]	N.D.[a]	N.D.[a]	N.D.[a]	N.D.[a]
Rad51-S2+S5S1	0.0105 ±0.0013	0.0289 ±0.0076	3.86 ± 1.3	N.D.[a]	N.D.[a]	N.D.[a]	N.D.[a]	N.D.[a]	N.D.[a]

[a]Not determined.

Each value reflects the average of three experiments. ± indicates standard deviation. S5S1, Swi5-Sfr1.

Supplementary Table 3. Dissociation and Hill constants of the Rad51 ssDNA and Rad51 dsDNA complexes

	WT	Rad51-L1	Rad51-L2	Rad51-S2
		ssDNA		
K_D (μM)	0.358 ± 0.025	0.664 ± 0.018	0.308 ± 0.019	0.895 ± 0.055
n	2.86 ± 0.29	2.94 ± 0.064	2.59 ± 0.11	2.25 ± 0.12
		dsDNA		
K_D (μM)	2.61 ± 0.20	N.D.[a]	3.52 ± 0.31	N.D.[a]
n	1.99 ± 0.091	N.D.[a]	1.80 ± 0.19	N.D.[a]

[a]Not determined.

Each value reflects the average of three experiments. ± indicates standard deviation.

Supplementary Table 4. Dissociation rate constant (k_{off}) values of Rad51 mutants for ssDNA

	[S5S1] per [Rad51]		
	0	0.1	0.5
WT	1.16 ± 0.055	0.269 ± 0.016	0.132 ± 0.019
Rad51-L1	1.79 ± 0.18	0.220 ± 0.026	0.181 ± 0.010
Rad51-L2	1.12 ± 0.041	0.647 ± 0.057	0.336 ± 0.061
Rad51-S2	1.46 ± 0.080	0.385 ± 0.028	0.210 ± 0.013

Each K_{off} value (10^{-2} [s^{-1}]) reflects the average of three experiments.

± indicates standard deviation. S5S1, Swi5-Sfr1.

Supplementary Table 5. Summary of FRET efficiencies for Fig. 5d and Supplementary Fig. 5a

	WT	Rad51-L1	Rad51-L2	Rad51-S2
		ATP		
E_{FRET} (ssDNA only)	0.450 ± 0.010	0.447 ± 0.0031	0.438 ± 0.0022	0.456 ± 0.0047
E_{FRET} (Rad51)	0.245 ± 0.0037	0.234 ± 0.0024	0.279 ± 0.0017	0.224 ± 0.0022
E_{FRET} (Rad51+S5S1)	0.161 ± 0.0033	0.164 ± 0.0035	0.152 ± 0.0029	0.173 ± 0.0016
		AMP-PNP		
E_{FRET} (ssDNA only)	0.441 ± 0.0010	0.437 ± 0.0015	0.451 ± 0.022	0.453 ± 0.0019
E_{FRET} (Rad51 only)	0.159 ± 0.0011	0.145 ± 0.00087	0.182 ± 0.0020	0.173 ± 0.00084
		No nucleotide		
E_{FRET} (ssDNA only)	0.443 ± 0.0021	N.D.[a]	N.D.[a]	N.D.[a]
E_{FRET} (Rad51 only)	0.347 ± 0.010	N.D.[a]	N.D.[a]	N.D.[a]

[a]Not determined.

Each value reflects the average of three experiments. ± indicates standard deviation. S5S1, Swi5-Sfr1.

Supplementary Table 6. Summary of FRET efficiencies for Fig. 6c and Supplementary Fig. 5c

	WT	L2
	ATP	
E_{FRET} (dsDNA only)	0.750 ± 0.0010	0.750 ± 0.00068
E_{FRET} (Rad51)	0.304 ± 0.0031	0.389 ± 0.0034
E_{FRET} (Rad51+S5S1)	0.286 ± 0.00090	0.373 ± 0.0022
	AMP-PNP	
E_{FRET} (dsDNA only)	0.740 ± 0.00065	0.740 ± 0.0026
E_{FRET} (Rad51)	0.247 ± 0.0014	0.348 ± 0.011
	No nucleotide	
E_{FRET} (dsDNA only)	0.739 ± 0.0010	N.D.[a]
E_{FRET} (Rad51)	0.558 ± 0.010	N.D.[a]

[a]Not determined.

Each value reflects the average of three experiments.

± indicates standard deviation. S5S1, Swi5-Sfr1.

Supplementary Table 7. Maximum quenching efficiency of 2AP for Fig. 4b,c and d

	Maximum quenching efficiency (Q_{max})
WT	0.369 ± 0.0030
WT + S5S1	0.423 ± 0.010
Rad51-L2	0.372 ± 0.010
Rad51-L2 + S5S1	0.392 ± 0.011

Each value reflects the average of three experiments.

± indicates standard deviation. S5S1, Swi5-Sfr1.

Supplementary Table 8. Constants used to calculate FRET efficiencies in Fig. 5d and Supplementary Fig. 5a

	ΦR per *ΦF*			
	WT	Rad51-L1	Rad51-L2	Rad51-S2
ssDNA only	0.778 ± 0.038	0.778 ± 0.038	0.778 ± 0.038	0.778 ± 0.038
Rad51+ ssDNA	0.867 ± 0.0054	0.813 ± 0.052	0.894 ± 0.014	0.850 ± 0.036
Rad51+S5S1+ ssDNA	0.891 ± 0.0036	0.912 ± 0.036	0.918 ± 0.017	0.859 ± 0.037

	I_{605} per I_{525}			
	WT	Rad51-L1	Rad51-L2	Rad51-S2
ssDNA only	0.0314 ± 0.0014	0.0314 ± 0.0014	0.0314 ± 0.0014	0.0314 ± 0.0014
Rad51+ ssDNA	0.0275 ± 0.0013	0.0301 ± 0.0012	0.0293 ± 0.0015	0.0302 ± 0.00040
Rad51+S5S1+ ssDNA	0.0238 ± 0.00074	0.0294 ± 0.0020	0.0259 ± 0.0011	0.0305 ± 0.0014

Each value reflects the average of three experiments. ± indicates standard deviation. S5S1, Swi5-Sfr1.

Supplementary Table 9. Constants used to calculate FRET efficiencies in Fig. 6c and Supplementary Fig. 5c

	ΦR per ΦF	
	WT	Rad51-L2
dsDNA only	0.362 ± 0.010	0.362 ± 0.010
Rad51+dsDNA	0.623 ± 0.019	0.669 ± 0.0032
Rad51+S5S1+dsDNA	0.607 ± 0.015	0.655 ± 0.0093

	I_{605} per I_{525}	
	WT	Rad51-L2
dsDNA only	0.0278 ± 0.0025	0.0278 ± 0.0025
Rad51+dsDNA	0.0276 ± 0.0015	0.0291 ± 0.0012
Rad51+S5S1+ dsDNA	0.0269 ± 0.0010	0.0268 ± 0.0019

Each value reflects the average of three experiments. ± indicates standard deviation. S5S1, Swi5-Sfr1.

Supplementary Table 10. Förster distance (R_0) and the overlap integral of the fluorescence emission spectrum of the donor and the absorption spectrum of the acceptor (J[λ]) of double-labeled ssDNA and dsDNA

	J(λ) x 10^{15} ($M^{-1}cm^{-1}nm^4$)	R_o (Å)
Double-labeled ssDNA	1.79	49.5
Double- labeled dsDNA	1.74	49.2

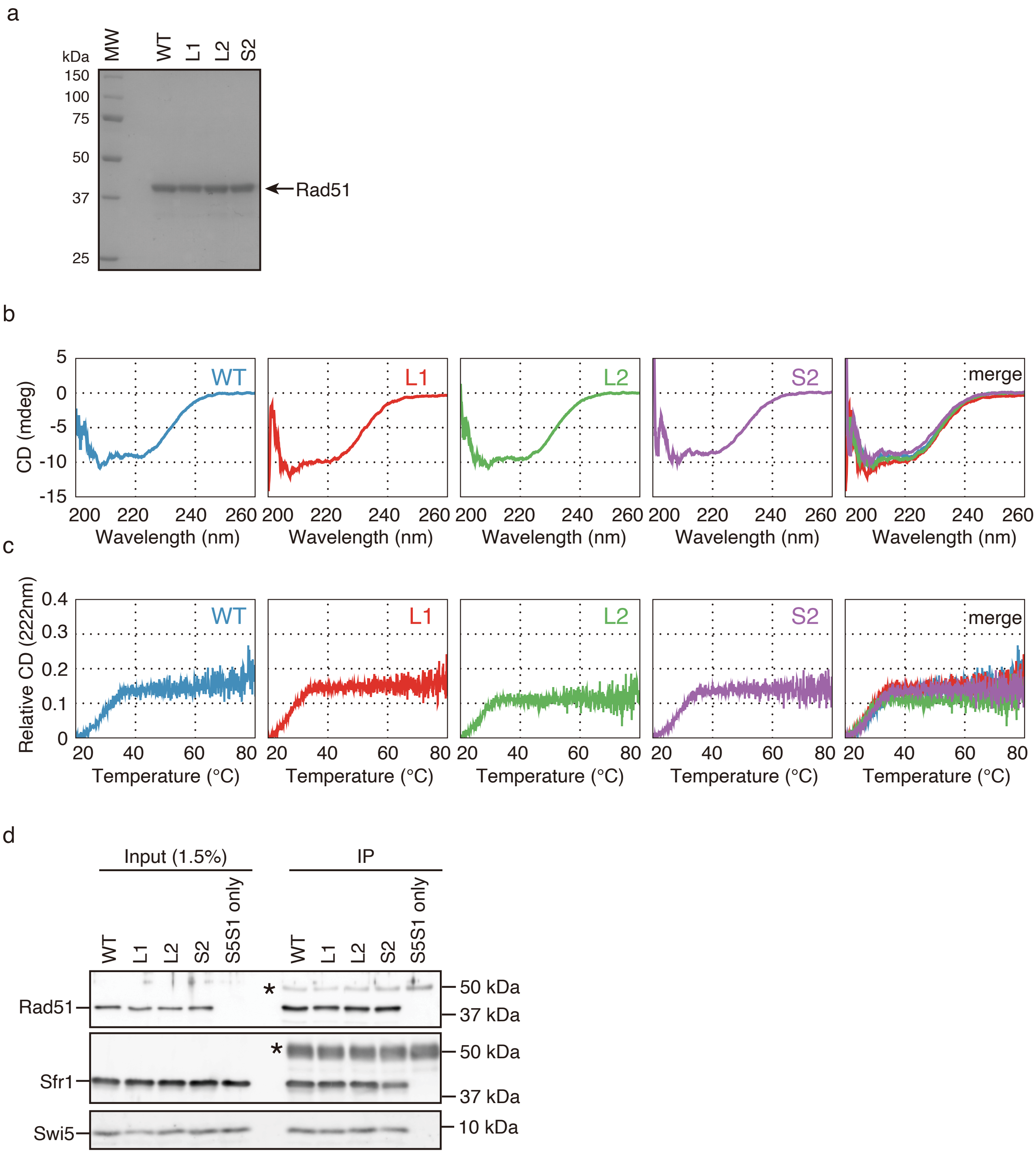

Supplementary Fig. 1. Wild-type and mutant Rad51 proteins used in this study. a Coomassie blue-stained SDS-PAGE of wild-type and mutant Rad51 proteins (Rad51-L1, Rad51-L2 and Rad51-S2) (1 µg/lane). **b** CD spectra of wild-type and mutant Rad51 proteins at 25 °C. CD spectra of Rad51 (0.1 mg/ml) in 150 µl of buffer (15 mM HEPES-KOH [pH 7.5], 1.5 mM $MgCl_2$, 0.5 mM DTT, 2.5 % [v/v] glycerol) in a 1 mm path-length quartz cuvette were measured at 200-

260 nm every 0.1 nm with a bandwidth of 2 nm using a J-820 spectropolarimeter (JASCO). All CD spectra are averages of four measurements and were corrected for with their respective buffer blanks. **c** Thermal stabilities of wild-type and mutant Rad51 proteins measured by CD at 222 nm. Spectra of Rad51 protein (0.1 mg/ml) in 200 µl of a buffer (7.5 mM HEPES--KOH [pH 7.5], 25 mM KCl, 0.75 mM $MgCl_2$, 0.25 mM DTT, 1.25 % [v/v] glycerol) were measured with a bandwidth of 10 nm every 0.1 °C using a JASCO J-820 spectropolarimeter. Temperature was changed from 20 to 80 °C and rate of increase in temperature was 2 °C/min. (Relative CD [222nm]) was calculated using the equation:

(Relative CD [222 nm]) = (CD at x°C) x (CD at 20°C)$^{-1}$ - 1

d Physical interactions between purified Rad51 mutants and Swi5–Sfr1 analyzed by a coimmunoprecipitation assay. After Rad51 (2.5 µM) and Swi5-Sfr1 (2.5 µM) were mixed for 30 min at 4 °C, affinity-purified rabbit anti-antibody Rad51 was added and the mixtures were incubated for further 1 h at 4 °C. Immunocomplexes pulled down were separated by SDS-PAGE, followed by western blotting using the indicated antibodies; rat anti-Rad51 (1:6000), rabbit anti-Sfr1 (1:3000), and rabbit anti-Swi5 (1:500), all of which are home-made[1] in Iwasaki's laboratory. Proteins were detected by a LAS-4000mini scanner (Fuji). The asterisks indicate IgG heavy chain. Source data are provided as a Source Data file.

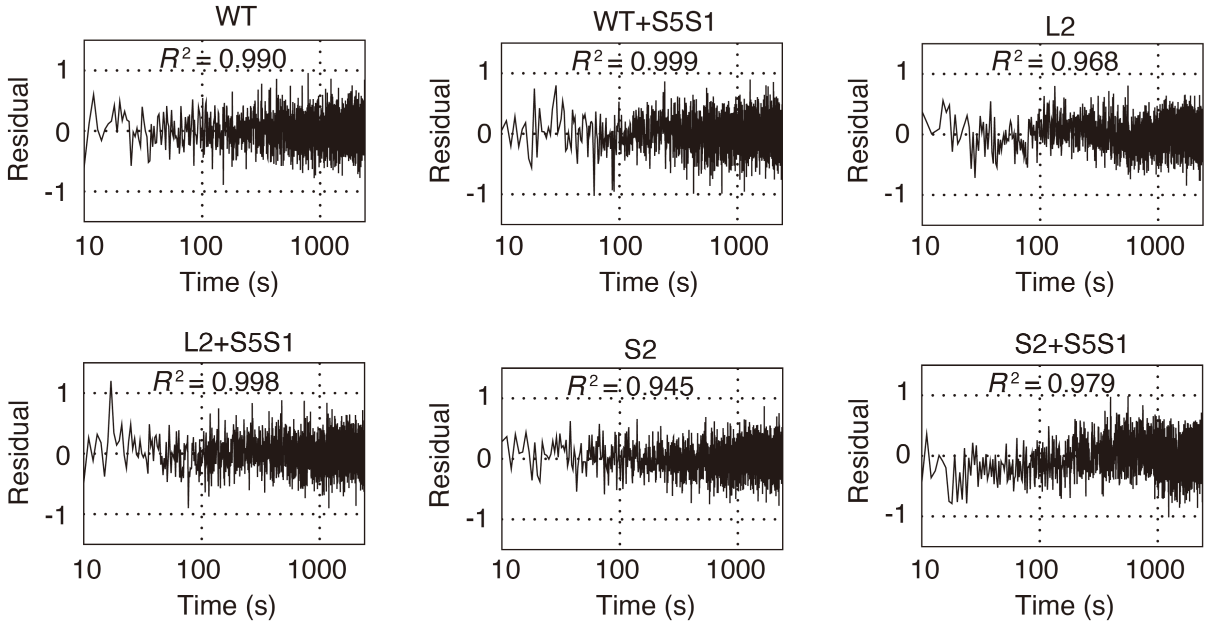

Supplementary Fig. 2. Residual analysis demonstrating the good fit of theoretical curves and experimental data. Residuals between experimental data of the DNA strand pairing assay in Fig. 2a and a theoretical curve obtained by simulation using DynaFit. Determination coefficients (R^2) are shown at the top of each graph. Source data are provided as a Source Data file.

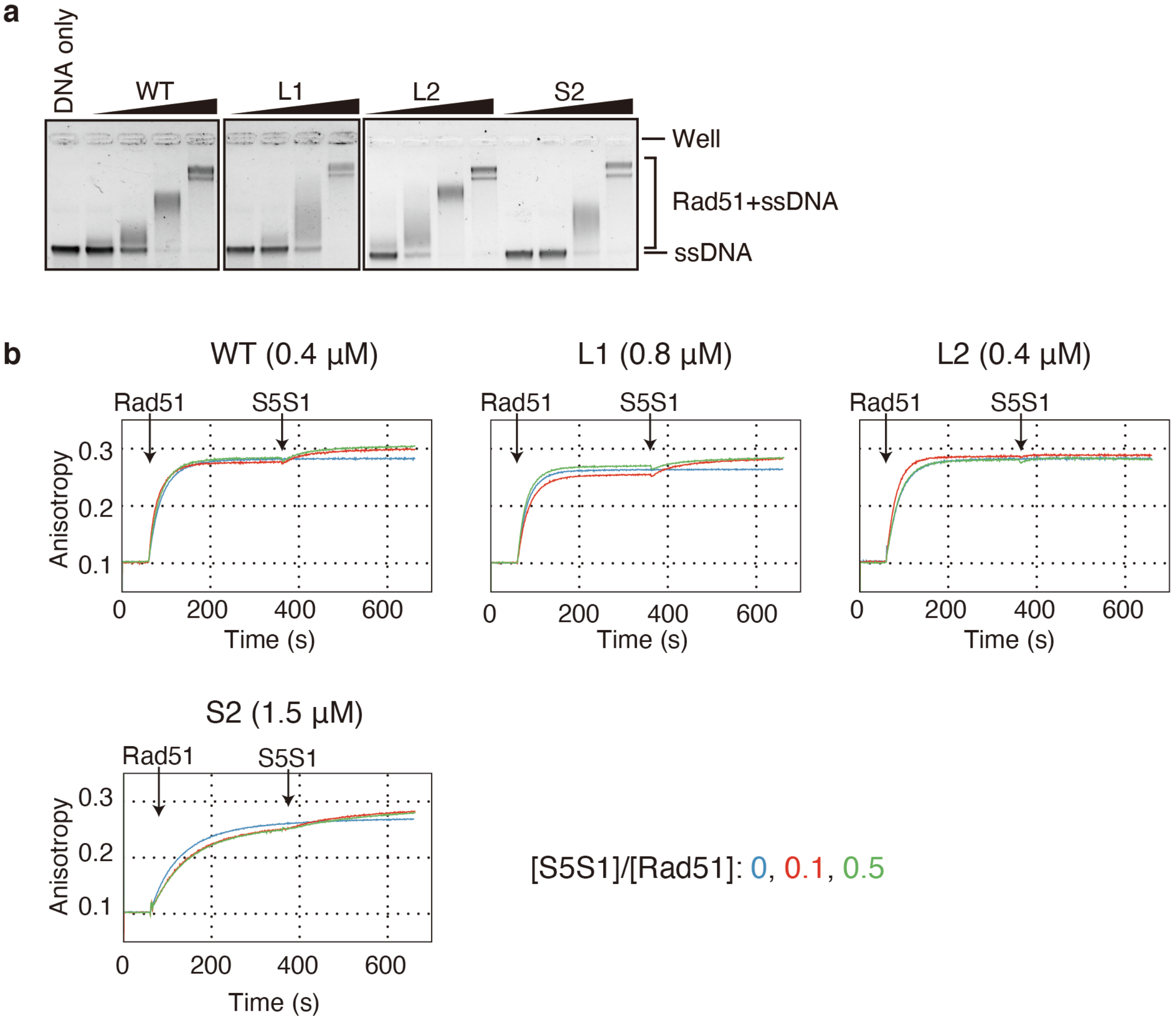

Supplementary Fig. 3. Gel shift assay and association kinetics of wild-type and mutant Rad51 proteins with ssDNA. a Wild-type and mutant Rad51 proteins (0.25, 0.5, 1.25, and 5 µM) were mixed with ssDNA (10 µM nucleotide). After incubation at 37°C for 15 min, Rad51-ssDNA filaments were cross-linked with glutaraldehyde and analyzed by 0.8% agarose gel electrophoresis. **b** The anisotropy of TAMRA-labeled ssDNA was monitored to detect association of wild-type Rad51 (upper left), Rad51-L1 (upper center), Rad51-L2 (upper right) or Rad51-S2 (lower left) with ssDNA at 25°C. After the indicated concentration of Rad51 protein was incubated with TAMRA-labeled ssDNA for 5 min, Swi5-Sfr1 was added at a Swi5-Sfr1:Rad51 ratio of 0 (blue lines), 0.1 (red lines), or 0.5 (green lines). Source data are provided as a Source Data file.

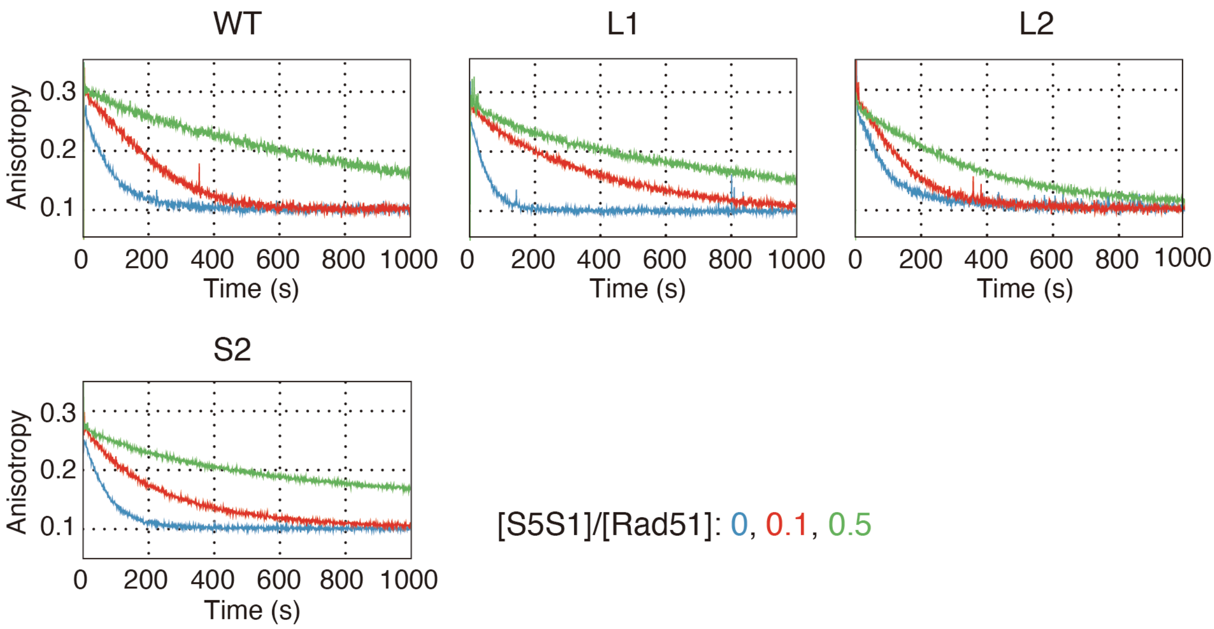

Supplementary Fig. 4. Dissociation kinetics of wild-type and mutant Rad51 proteins from ssDNA. The association complex prepared in Supplementary Fig. 3b was diluted 1:40 with reaction buffer (time=0) and fluorescence anisotropy was measured continuously for wild-type Rad51 (upper left), Rad51-L1 (upper center), Rad51-L2 (upper right) or Rad51-S2 (lower left). Swi5-Sfr1 was added at a Swi5–Sfr1:Rad51 ratio of 0 (blue lines), 0.1 (red lines), or 0.5 (green lines). Source data are provided as a Source Data file.

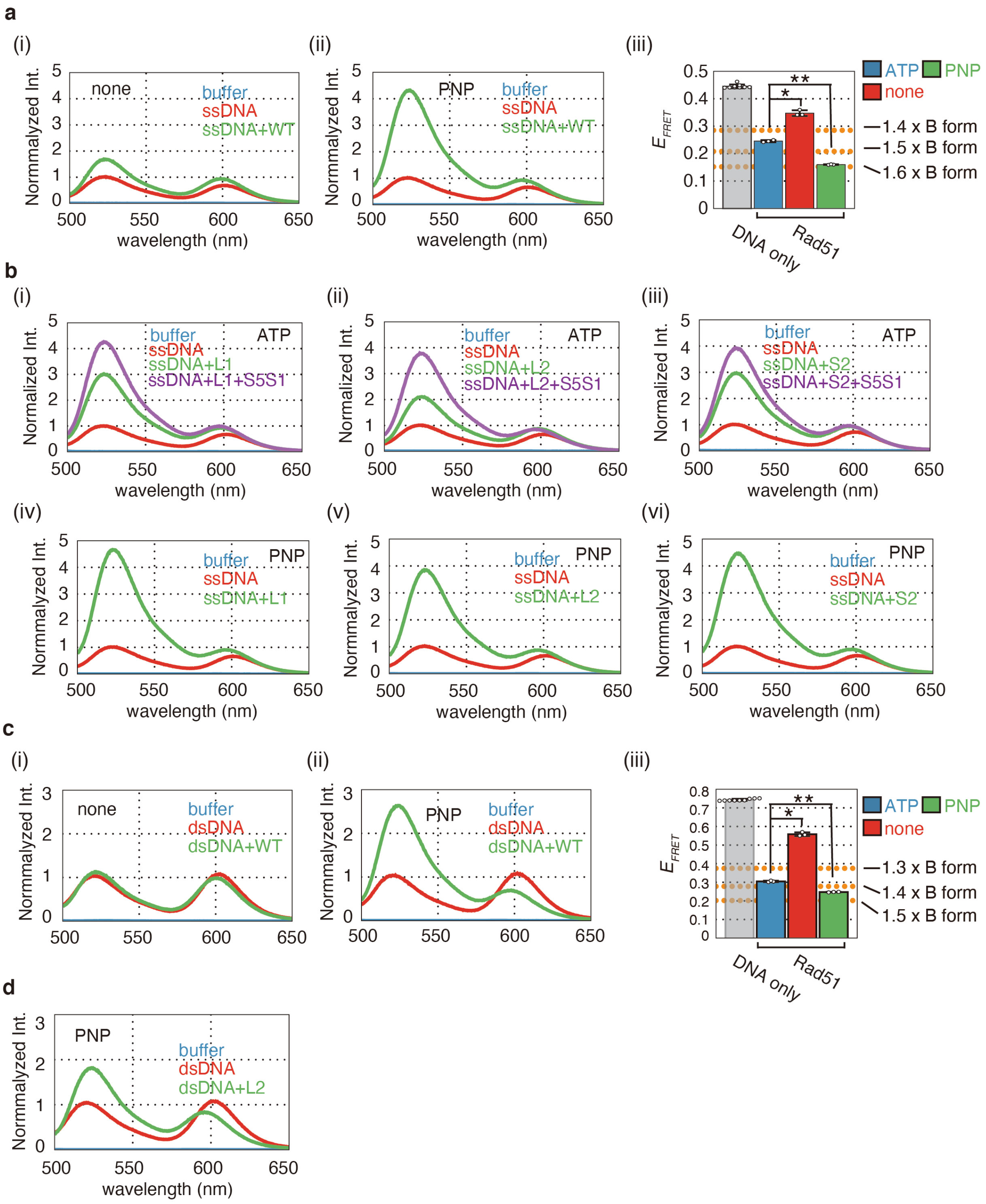

Supplementary Fig. 5. Emission spectra of ssDNA and dsDNA double-labeled with fluorescein and rhodamine. a Nucleotide dependency of ssDNA elongation by Rad51. Wild-

type Rad51 (3 µM) was mixed with the double-labeled ssDNA (3 µM nucleotides) and the emission spectra of fluorescein and rhodamine were collected following excitation at 493 nm, as described in *Methods*. (i) Without nucleotide and (ii) with AMP-PNP. (iii) The FRET efficiency (E_{FRET}) of each reaction without nucleotide, with ATP or with AMP-PNP, was calculated from a-(i), a-(ii) and Fig. 5d. The data for reactions containing ATP and AMP-PNP were originally presented in Fig. 5d and is included here purely for the purpose of comparison. Orange dotted-lines show the distances between two fluorophores compared to B-form DNA. * p = 9.14 x 10^{-5} and ** p = 3.04 x 10^{-6} by two-tailed Student's t test. Data are expressed as the mean ± s.d. (n = 3 independent experiments). Source data are provided as a Source Data file. **b** ssDNA elongation by Rad51 mutant proteins in the presence of ATP or AMP-PNP. (i) Rad51-L1 with ATP, (ii) Rad51-L2 with ATP, (iii) Rad51-S2 with ATP, (iv) Rad51-L1 with AMP-PNP, (v) Rad51-L2 with AMP-PNP (vi) Rad51-S2 with AMP-PNP. **c** Nucleotide dependency of dsDNA elongation by Rad51. Wild-type protein (8 µM) was mixed with the double-labeled dsDNA (3 µM bp) and the emission spectra of fluorescein and rhodamine were collected following excitation at 493 nm, as described in Methods. (i) Without nucleotide and (ii) with AMP-PNP. (iii) The FRET efficiency (E_{FRET}) of each reaction condition without nucleotide, with ATP or with AMP-PNP, was calculated from c-(i), c-(ii), and Fig. 6c. The data for reactions containing ATP and AMP-PNP were originally presented in Fig. 5d and is included here purely for the purpose of comparison. Orange dotted-lines show the distances between two fluorophores compared with B-form DNA. * p = 2.25.14 x 10^{-6} and ** p = 1.76 x 10^{-5} by two-tailed Student's t test. **d** dsDNA elongation by Rad51-L2 in the presence of AMP-PNP. Data are expressed as the mean ± s.d. (n = 3 independent experiments). Source data are provided as a Source Data file. Source data are provided as a Source Data file.

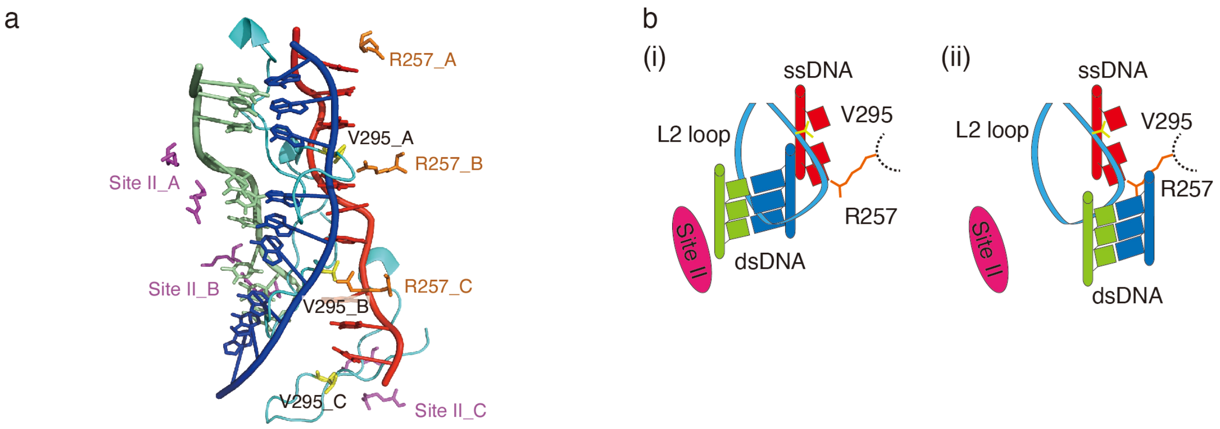

Supplementary Fig. 6. C1 structural model showing a collision between L2 and dsDNA. a In this model, Arg-257 in L1 inserts into the inter-triplet gap of dsDNA and site II is oriented to interact with dsDNA. The resultant structure shows a collision between L2 and the donor dsDNA. The initial ssDNA is shown in red, donor dsDNA in green and blue, Arg-257 in L1 is in orange, L2 is in cyan, Val-295 in L2 is in yellow and site II is in magenta. **b** Schematic of the possible configurations to avoid a collision between L2 and dsDNA. (i) One strand of the dsDNA passes through L2 loop or (ii) the dsDNA is moved away from L2. Source data are provided as a Source Data file.

Supplementary Reference

1. Haruta N., *et al.* The Swi5-Sfr1 complex stimulates Rhp51/Rad51- and Dmc1-mediated DNA strand exchange in vitro. *Nat Struct Mol Biol* **13**, 823-830 (2006).
