# Supplementary material for: Real-time tracking reveals catalytic roles for the two DNA binding sites of Rad51
Source: Nat Commun. 2020 Jun 11;11:2950. doi: 10.1038/s41467-020-16750-3 (PMC7289862; doi:10.1038/s41467-020-16750-3)
Supplement: Supplementary file 3 — Reporting Summary [file 41467_2020_16750_MOESM3_ESM.pdf]

nature research

Corresponding author(s): Hiroshi Iwasaki

Last updated by author(s): May 7, 2020

Reporting Summary

Nature Research wishes to improve the reproducibility of the work that we publish. This form provides structure for consistency and transparency in reporting. For further information on Nature Research policies, see Authors & Referees and the Editorial Policy Checklist.

Statistics

For all statistical analyses, confirm that the following items are present in the figure legend, table legend, main text, or Methods section.

n/a	Confirmed	
☐	☒	The exact sample size (*n*) for each experimental group/condition, given as a discrete number and unit of measurement
☐	☒	A statement on whether measurements were taken from distinct samples or whether the same sample was measured repeatedly
☐	☒	The statistical test(s) used AND whether they are one- or two-sided<br>*Only common tests should be described solely by name; describe more complex techniques in the Methods section.*
☒	☐	A description of all covariates tested
☒	☐	A description of any assumptions or corrections, such as tests of normality and adjustment for multiple comparisons
☐	☒	A full description of the statistical parameters including central tendency (e.g. means) or other basic estimates (e.g. regression coefficient) AND variation (e.g. standard deviation) or associated estimates of uncertainty (e.g. confidence intervals)
☐	☒	For null hypothesis testing, the test statistic (e.g. *F*, *t*, *r*) with confidence intervals, effect sizes, degrees of freedom and *P* value noted<br>*Give P values as exact values whenever suitable.*
☒	☐	For Bayesian analysis, information on the choice of priors and Markov chain Monte Carlo settings
☒	☐	For hierarchical and complex designs, identification of the appropriate level for tests and full reporting of outcomes
☒	☐	Estimates of effect sizes (e.g. Cohen's *d*, Pearson's *r*), indicating how they were calculated

Our web collection on statistics for biologists contains articles on many of the points above.

Software and code

Policy information about availability of computer code

Data collection	N/A. We did not use any software for data collection.
Data analysis	MODELLER software ver. 9.17 was used for structural model constructions.<br>DynaFit ver 4.08.016 was used for kinetic analysis of DNA strand exchange.<br>Kaleida Graph ver 4.5.1 was used for calculations of Kd (ATP, ssDNA, and dsDNA) of Koff (Rad51 from ssDNA)

For manuscripts utilizing custom algorithms or software that are central to the research but not yet described in published literature, software must be made available to editors/reviewers. We strongly encourage code deposition in a community repository (e.g. GitHub). See the Nature Research guidelines for submitting code & software for further information.

Data

Policy information about availability of data

All manuscripts must include a data availability statement. This statement should provide the following information, where applicable:

- Accession codes, unique identifiers, or web links for publicly available datasets
- A list of figures that have associated raw data
- A description of any restrictions on data availability

The atomic coordinates (5H1B and 5H1C) have been deposited in the Protein Data Bank (www.wwpdb.org). All other data are available from the corresponding author on reasonable request. The model structures have been submitted to the Biological Structure Model Archive (BSM-Arc) under BSM-ID BSM00017 (https://bsma.pdbj.org/entry/17). All other data supporting the findings of this study are available within the paper and its Supplementary Information files. Any further information can be obtained from the authors upon reasonable request.

Field-specific reporting

Please select the one below that is the best fit for your research. If you are not sure, read the appropriate sections before making your selection.

☒ Life sciences ☐ Behavioural & social sciences ☐ Ecological, evolutionary & environmental sciences

For a reference copy of the document with all sections, see nature.com/documents/nr-reporting-summary-flat.pdf

Life sciences study design

All studies must disclose on these points even when the disclosure is negative.

Sample size	No statistical methods were used to determine sample size. Sample size number was determined empirically from past experience based on previous results obtained with methods & approaches. The norm in biochemical experiments is to repeat the experiment for a total of at least two times for qualitative analysis, or at least three times for quantitative analysis in which the mean will be plotted. All of our experiments adhere to these accepted standards.
Data exclusions	We did not exclude any data.
Replication	Independent experiments were repeated at least two times for qualitative analysis, or at least three times for quantitative analysis.
Randomization	Randomization is not applicable for the biochemical and structural work presented. This study only involved biochemical analysis where the activity of purified proteins was monitored. There was no experimental sampling that could introduce bias. As such, there is no need for randomization.
Blinding	Blinding is not relevant for the presented work and is not necessary as samples were not allocated to groups.

Reporting for specific materials, systems and methods

We require information from authors about some types of materials, experimental systems and methods used in many studies. Here, indicate whether each material, system or method listed is relevant to your study. If you are not sure if a list item applies to your research, read the appropriate section before selecting a response.

Materials & experimental systems

n/a	Involved in the study	
☐	☒	Antibodies
☒	☐	Eukaryotic cell lines
☒	☐	Palaeontology
☒	☐	Animals and other organisms
☒	☐	Human research participants
☒	☐	Clinical data

Methods

n/a	Involved in the study	
☒	☐	ChIP-seq
☒	☐	Flow cytometry
☒	☐	MRI-based neuroimaging

Antibodies

Antibodies used	Rabbit anti-Rad51 is a home-made polyclonal antibody against purified S. pombe Rad51. Five µl for one co-immunocomplex formation in 100 µL were used. Rat anti-Rad51 is a home-made polyclonal antibody against purified S. pombe Rad51 (1:6000 for western blot). Rabbit anti-Sfr1 is a home-made polyclonal antibody against purified S. pombe Sfr1 (1:3000 for western blot). Rabbit anti-Swi5 is a home-made polyclonal antibody against purified S. pombe Swi5 (1:500 for western blot). Anti-rabbit IgG-HRP from donkey (1:5000, NA934) and anti-rat IgG-HRP (1:5000, 12-035-153) from donkey used for 2nd antibody for western blotting were from GE Healthcare and Jackson Laboratories, respectively. *Haruta et al (Nature struct Mol Biol 13, 823 (2006)) DOI: 10.1038/nsmb1136.
Validation	All home-made antibodies (anti-Rad51, anti-Sfr1, and anti-Swi5) were all validated using S. pombe strains with a deletion mutation of the corresponding gene in Haruta et al*. GE Healthcare and Jackson Laboratories validated the anti-rabbit IgG-HRP (NA934) and anti-rat IgG-HRP (712-035-153) for the detection of rabbit IgG and rat IgG, as stated on the website, respectively. *Haruta et al Nat Struct Mol Biol 2006: doi:10.1038/nsmb1136.
